# Simulation of charge transport in pixelated CdTe

**DOI:** 10.1088/1748-0221/9/12/C12027

**Published:** 2014-12-01

**Authors:** M. Kolstein, G. Ariño, M. Chmeissani, G. De Lorenzo

**Affiliations:** Institut de Física d’Altes Energies (IFAE), Edifici Cn, Universitat Autónoma de Barcelona (UAB), E-08193 Bellaterra, Spain

**Keywords:** Charge transport and multiplication in solid media, Detector modelling and simulations II (electric fields, charge transport, multiplication and induction, pulse formation, electron emission, etc), Charge induction, Solid state detectors

## Abstract

The Voxel Imaging PET (VIP) Pathfinder project intends to show the advantages of using pixelated semiconductor technology for nuclear medicine applications to achieve an improved image reconstruction without efficiency loss. It proposes designs for Positron Emission Tomography (PET), Positron Emission Mammography (PEM) and Compton gamma camera detectors with a large number of signal channels (of the order of 10^6^). The design is based on the use of a pixelated CdTe Schottky detector to have optimal energy and spatial resolution. An individual read-out channel is dedicated for each detector voxel of size 1 × 1 × 2 mm^3^ using an application-specific integrated circuit (ASIC) which the VIP project has designed, developed and is currently evaluating experimentally.

The behaviour of the signal charge carriers in CdTe should be well understood because it has an impact on the performance of the readout channels. For this purpose the Finite Element Method (FEM) Multiphysics COMSOL software package has been used to simulate the behaviour of signal charge carriers in CdTe and extract values for the expected charge sharing depending on the impact point and bias voltage. The results on charge sharing obtained with COMSOL are combined with GAMOS, a Geant based particle tracking Monte Carlo software package, to get a full evaluation of the amount of charge sharing in pixelated CdTe for different gamma impact points.

## 1 Introduction

The Voxel Imaging PET (VIP) Pathfinder project^1^ aims to show that the VIP design for PET [1] allows for better image reconstruction because of the excellent spatial and energy resolution it can provide, compared to state-of-the-art crystal PETs. This is obtained by using finely segmented CdTe allowing for precise precision measurement of the gamma impact point with an excellent energy resolution of about 1% at 511 keV [2]. However, the drawback of small pixel sizes is that a large fraction of photons have energy depositions in more than one neighbouring pixels. To correct for this, either charge sharing correction algorithms should be studied or the charge sharing events should be rejected, so it is important to know the amount of charge sharing events. Following the example of other experiments (e.g. [3–5]), we used a tracking program (the Geant4-based Architecture for Medicine-Oriented Simulations (GAMOS) software [6]) to estimate the size of the initial charge carrier cloud and, subsequently, the finite element methods (FEM) software package COMSOL [7] to numerically calculate the behaviour of the charge carriers in the detector and the resulting charge induction. The convolution of the results from these programs gives an estimate of the total amount of charge sharing.

## 2 Theory and simulation model

### Charge transport, convection and diffusion

An incoming gamma will create a cloud of charge carriers (electrons and holes in equal numbers) in the semi-conductor material. The number of charge carriers is proportional to the energy of the gamma. When an electric bias potential *ϕ* is applied to the detector, the charge carriers move towards the oppositely charged electrodes, creating an electrical signal that is amplified and measured. The value for the electric potential within the semi-conductor is obtained by solving the Laplace equation: ∇^2^*ϕ* = 0 while keeping *ϕ* = 0 V at the anodes and *ϕ* = −2000 V at the cathodes and keeping all in- and outgoing fluxes to 0. For the drift velocity of the charge carriers we have ${\vec{\nu }}_{\mathrm{drift},e,h}=-\mu_{e,h}\cdot \vec{\nabla }\phi$, where *μ*_*e*_, *μ*_*h*_ are the mobilities for electrons and holes respectively. The probability for electrons and holes to get trapped while drifting towards the electrodes is expressed by their lifetimes, τ_*e*_, τ_*h*_. In table 1 the main properties for electrons and holes in CdTe are summarized. The mean free path (the product of *μ* and τ) is smaller for holes than for electrons, since holes are more affected by trapping. The density of electrons $n(\vec{r},t)$ and holes $p(\vec{r},t)$ in the semi-conductor as a function of time and position is solved by the convection and diffusion equations:

(2.1) $$\frac{dn}{dt}+\nabla \cdot (\mu_{e}\cdot n\cdot \nabla \phi )-\nabla \cdot (D_{e}\nabla n)+\frac{n}{\tau_{e}}=G_{e},$$

(2.2) $$\frac{dp}{dt}-\nabla \cdot (\mu_{h}\cdot p\cdot \nabla \phi )-\nabla \cdot (D_{h}\nabla p)+\frac{p}{\tau_{h}}=G_{h},$$

where $D_{e,h}=\frac{k_{B}\cdot T}{q}\cdot \mu_{e,h}$, corresponds to the diffusion coefficient, the terms $\frac{n}{\tau_{e}}$, $\frac{p}{\tau_{h}}$ correspond to the trapping of the charge carriers, and *G_e,h_* corresponds to the generation term which is equal to $\delta (\vec{r}-\vec{r^{\prime }})\delta (t-t^{\prime })$, with $\vec{r^{\prime }}$ and *t*′ the location and time of the impact of the gamma.

### Charge induction

The measured signal from the detector comes from charge induction which is caused by the motion of the charge carriers and is proportional to the energy deposition of the original incoming gamma. Charge induction starts from the moment the charge carriers are created until they are all collected. A method to calculate charge induction was found independently by both Shockley [9] and Ramo [10], using so-called *weighting potentials* to simplify the calculation of charge induction. The induced charge *Q_k_* on an electrode *k* by a single charge carrier *q* depends on the weighting potential *Ψ_k_* at the start *z*_0_ and at the end point *z*_1_ of the charge carrier trajectory as: *Q_k_* = −*q*(*Ψ*_*k*_(*z*_1_)−*Ψ*_k_(*z*_0_)). The total induced charge is equal to the sum of the induced charges by all electrons and holes. The weighting potential *Ψ*_*k*_ for anode *k* can be obtained numerically by solving the Laplace equation: ∇^2^*Ψ*_*k*_ = 0, where *Ψ*_*k*_ is set to 1 V at anode *k* and 0 V at all other electrodes and all in- and outgoing fluxes are set to 0. In the case of planar electrodes (i.e., where the lateral size of the electrodes is larger than the detector thickness *d*), the weighting potential reduces to a straightforward linear function of the depth of interaction *z* and can be calculated analytically with *ϕ*_*w*_ = *z*/*d*. When a charge cloud is created far away from the anode, electrons will move towards the anode for a longer time and thus induce more charge than electrons created near the anode, whereas holes will induce relatively little charge. The opposite argument holds when the charge cloud is created near the cathode, in which most of the induced charge comes from the holes. In both cases the total sum of induced charge is 1, independent of the gamma impact point, as long as there is no trapping. Charge carriers can get trapped by atoms in the semi-conductor, so the charge carrier density is not constant and the total induced charge *Q_k_* will be smaller than the original charge *Q*_0_ created by the gamma impact. Because the probability to get trapped is bigger for holes than for electrons, the signal gets worse when the impact point is further away from the cathode. For planar electrodes, charge induction depends linearly on *z*, so we could use the Hecht equation [11] to calculate the effect of trapping.

### Small pixel effect

**Figure 1:**
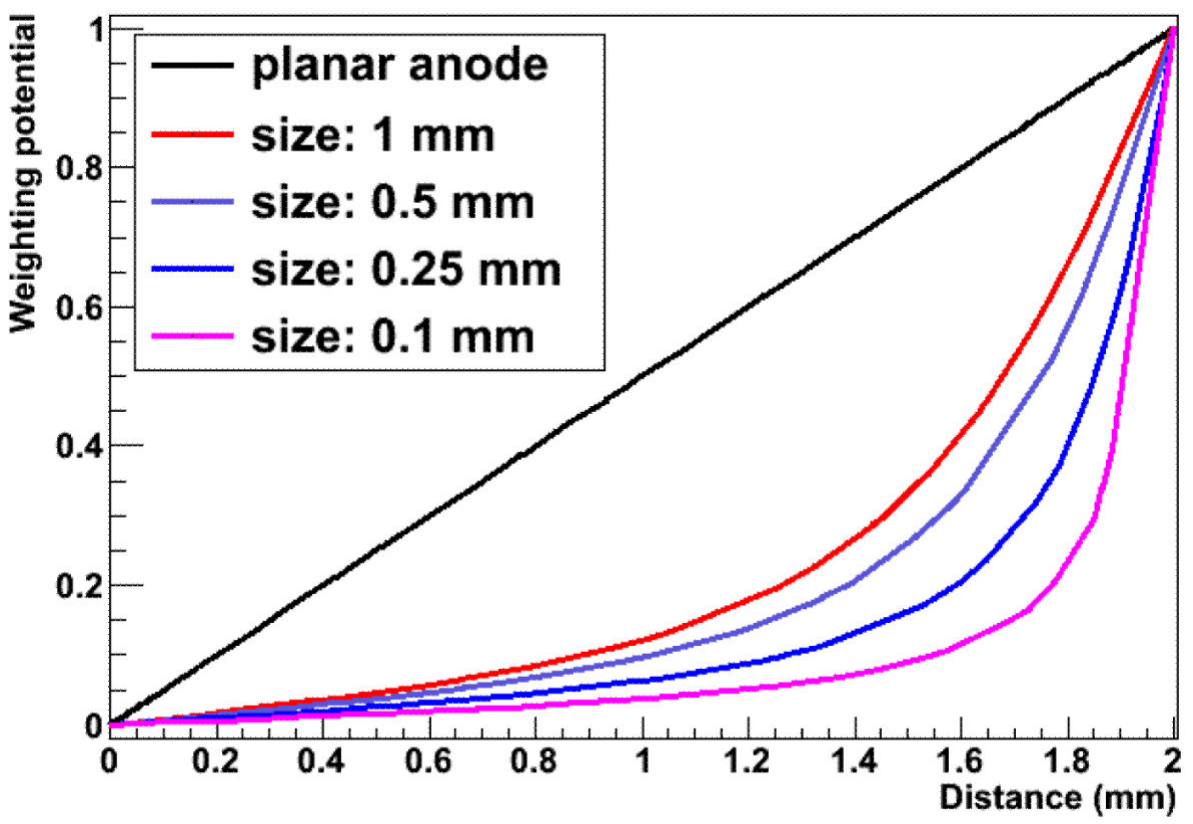
Weighting potentials for different lateral pixel sizes, with a pixel thickness of 2 mm. With a large pixel size, the weighting potential goes linear with *z*. For small pixels, the weighting potential has a steep rise near the anode.

With a finely segmented detector, with pixel sizes of 1 × 1 mm^2^, the spatial resolution of the detector will improve. In this case, where the pixel lateral size is small compared to the pixel thickness (2 mm), the *small pixel effect* will occur, where the dependence of the weighting potential on the depth of interaction is no longer linear (see figure 1) and the Hecht equation is no longer valid to account for trapping. In this case, the charge induction mainly depends on the electron contribution, and hence, is less affected by trapping. Additionally, when decreasing the pixel pitch, there will be more charge sharing between neighbouring pixels.

### Charge Induction Efficiency

The Charge Induction Efficiency (CIE) is defined by the induced charge *Q_k_* at a certain pixel anode *k* divided by the total initial charge of the charge cloud *Q*_0_, where *Q_k_* is given by:

(2.3) $$Q_{k}=-q\int_{t_{0}}^{t_{1}}\;dt\;\int_{\Omega }d\Omega \cdot c(\vec{r},t)\cdot \mu_{c}\cdot \vec{\nabla }\phi \cdot \vec{\nabla }\psi_{k},$$

with Ω the volume of the semi conductor, $c(\vec{r},t)$ the concentration of charge carriers (i.e. the number of electrons *n* or holes *p* as obtained from (2.1) and (2.2)) as a function of position and time, and *μ*_*c*_ the charge carrier mobility. Because the non-linearity of the weighting potential, this equation can only be calculated numerically. In principle one could calculate *Q_k_* for a particular position of the impact point after a certain moment of time *t*_1_, and then repeat this calculation for all possible positions of the impact point in the detector, for electrons and holes seperately. Since this would be very time consuming, we take advantage of the adjoint method, as described by Prettyman [12, 13].

Whereas *n* and *p* from the continuity equations (2.1) and (2.2) correspond to the number of charge carriers, with the adjoint approach we have adjoint variables *n*^+^ and *p*^+^ which represent the CIE for electrons and holes respectively, when the adjoint generation term is defined as *G_c_*^+^ = *μ*_*c*_∇_*ϕ*_ ∇_Ψ*k*_. By solving the adjoint equations, we inmediately obtain the CIE for electrons and holes for all possible gamma impact points in the detector as a function of time. The complete adjoint equations to be solved for electrons and holes are:

(2.4) $$\frac{dn^{+}}{dt}-\nabla \cdot (\mu_{e}\cdot n^{+}\cdot \nabla \phi )-\nabla \cdot (D_{e}\nabla n^{+})+\frac{n^{+}}{\tau_{e}}=\mu_{e}\nabla \phi \nabla \psi_{k}.$$

(2.5) $$\frac{dp^{+}}{dt}+\nabla \cdot (\mu_{h}\cdot p^{+}\cdot \nabla \phi )-\nabla \cdot (D_{h}\nabla p^{+})+\frac{p^{+}}{\tau_{h}}=\mu_{h}\nabla \phi \nabla \psi_{k}.$$

Note that the sign is reversed on the drift term, compared with equations (2.1) and (2.2). A more detailed explanation of the adjoint approach can be found in [14].

## 3 Results

Unless otherwise stated, for all results a bias voltage of −2000 V was used and CdTe was characterized in COMSOL by the parameters from table 1. The values for the mobilities and lifetimes used in this analysis were confirmed by experimental data [15]. An array of 3 × 3 CdTe detectors with pixel pitch of 1 mm and thickness 2 mm was simulated and the CIE was obtained with COMSOL by the following steps:

- Solve the Laplace equation to obtain the electric field potential *ϕ*.
- Solve the Laplace equation to obtain the weighting field potential *Ψ*_*k*_.
- Solve the adjoint equations (2.4) and (2.5) to obtain the CIE for electrons and holes for all possible impact points, as a function of time.

**Figure 2:**
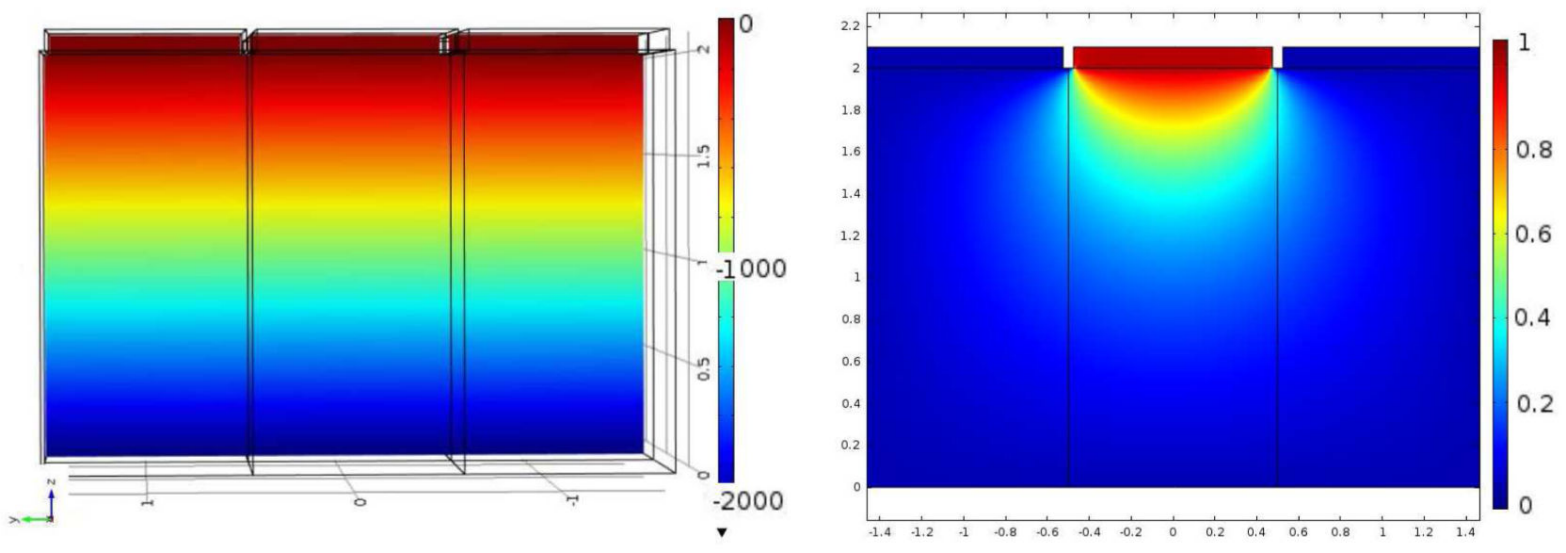
The bias voltage *ϕ* and the weighting potential *Ψ*_*k*_ for anode *k* along the XZ plane.

**Figure 3:**
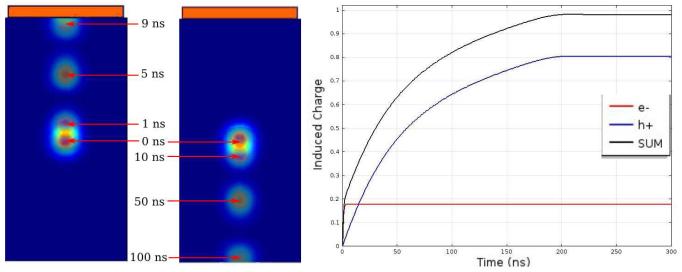
The figure on the *left* shows the electron and hole density as a function of time, obtained by solving continuity equations (2.1) and (2.2). When plugging the densities into (2.3) we obtain the CIE for a particular impact point versus time. The plot on the *right* shows the CIE with impact point *z* = 1.9 mm, i.e., very near the anode so the signal mainly depends on holes.

**Figure 4:**
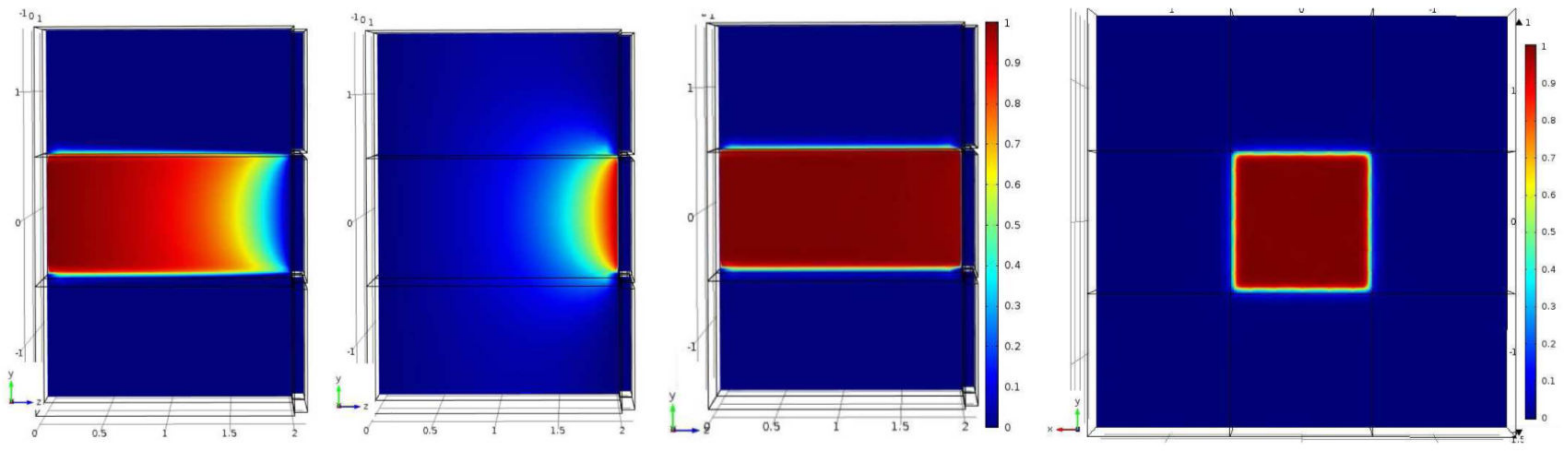
*Most left:* CIE for electrons only. *2nd:* CIE for holes only. *3th and most right:* total CIE in the ZY plane with X = 0 and the XY plane with Z = 1, respectively.

Figure 2 shows the electric potential and the weighting potential. The small pixel effect is illustrated by the steep rise of the weighting potential near the anode. Figure 3 shows the electron and hole cloud positions as a function of time. Electrons will move 1 mm every 9.1 ns, and holes 1 mm every 100 ns, as expected from the given values for the mobilities and the bias voltage. The result of the numerical calculation of the charge induction by using equation (2.3) in COMSOL is shown on the right in figure 3. As explained in the previous section, doing this calculation for all possible impact points within the detector would take an impossible amount of time and effort. Instead, we use COMSOL to simultaneously calculate all values of the CIE for all possible impact points as a function of time by solving the adjoint continuity equations (2.4) and (2.5). From figure 4 we can see that the total CIE is close to 1 over the entire region of the pixel and only decreases near the edges of neighbouring pixels where charge sharing sets in.

**Figure 5:**
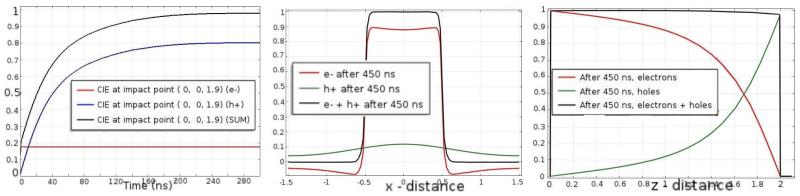
*Left:* CIE versus time for an impact point near the anode, with *z* = 1.9 mm. Note that the curves are identical to those on the right in figure 3. After 300 ns, all holes have reached the opposite side of the detector. *Centre:* CIE versus lateral direction of the impact point with *z* = 0, after 450 ns. *Right:* The CIE versus the interaction depth of the impact point with x and y at the center of the pixel, after 450 ns. For most impact points the total CIE is close to 1, only near the pixel edges charge sharing will set in.

Once we have obtained the CIE as a function of all possible impact points and for all times, we can plot it versus time for particular impact points or versus distance for a particular time. Figure 5 shows the CIE as a function of time, lateral position (x-axis), and impact point depth of interaction (z-axis), from left to right respectively.

**Figure 6:**
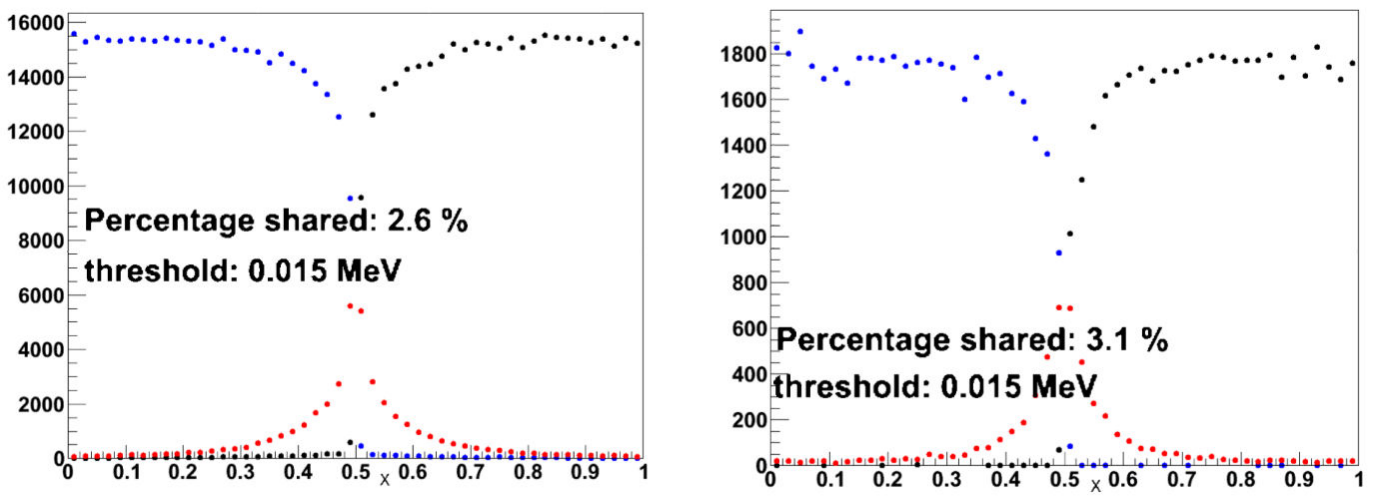
Charge sharing between two neighbouring pixels due only to the size of the initial charge clouds with (*left:*) 122 keV gammas and (*right:*) 511 keV gammas. Each dot indicates a gamma impact point versus the lateral position. The blue dots indicate events with more than 15 keV deposited in the left pixel. The blacks dots indicate events with more than 15 keV deposited in the right pixel. The red dots indicate events with energy depositions of more than 15 keV in both pixels.

**Figure 7:**
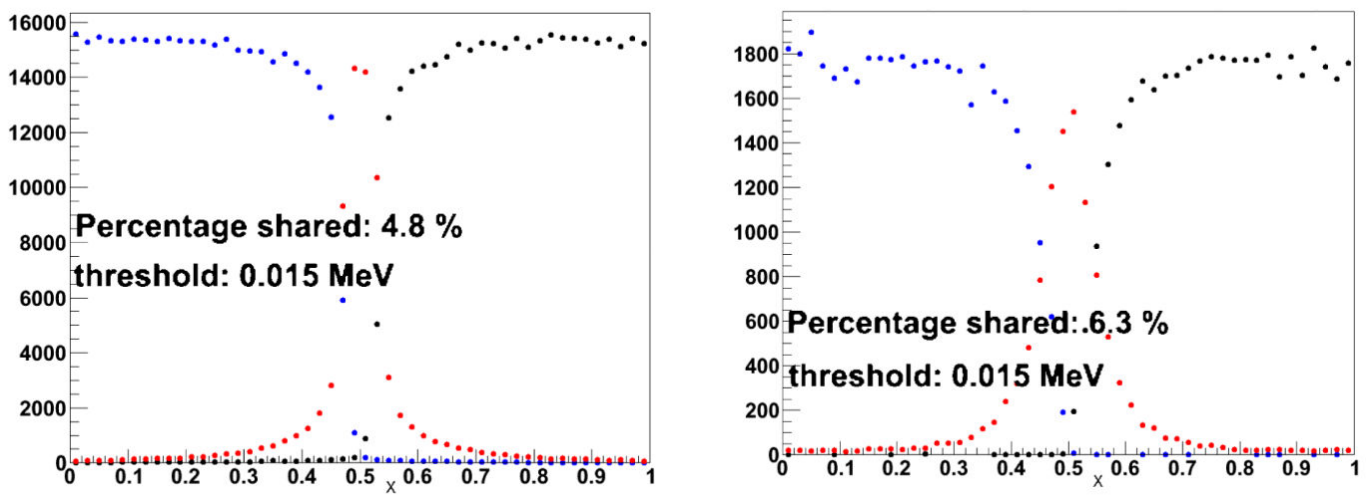
Total charge sharing between two neighbouring pixels with (*left:*) 122 keV gammas and (*right:*) 511 keV gammas. As in figure 6, each dot indicates a gamma impact point versus the lateral position.

**Figure 8:**
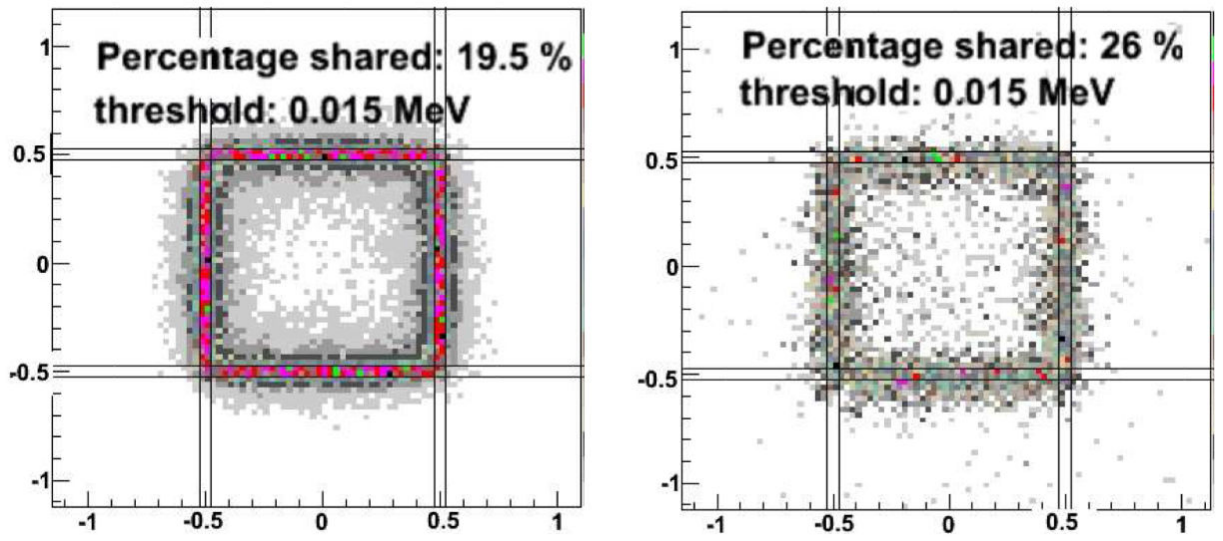
Charge sharing between one center pixel and eight neighbouring pixels with (*left:*) 122 keV gammas and (*right:*) 511 keV gammas. Plotted are the original impact points of events that had energy depositions of more than 15 keV in the center pixel and at least one neighbouring pixel.

Figure 6 shows the amount of charge sharing between two neighbouring cells due only to the sizes of the charge clouds (i.e., without the convection and diffusion as simulated by COMSOL) for gammas of 122 keV and 511 keV and a threshold of 15 keV. Figure 7 shows the total amount of charge sharing between two neighbouring cells, by applying the corresponding CIE obtained with COMSOL to each of the charge carrier positions in the original charge cloud as obtained by GAMOS. Figure 8 shows that the total charge sharing for a 3 × 3 array set-up for 122 keV and 511 keV gammas and with a 15 keV threshold is 19.5% and 26% respectively. The average number of charge sharing pixels is 2 with 122 keV and 2.1 with 511 keV. With a threshold of 5 keV, in a 3 × 3 array, the charge sharing would be 31.3% (122 keV) and 35.6% (511 keV), and the average number of firing pixels is still 2.

**Figure 9:**
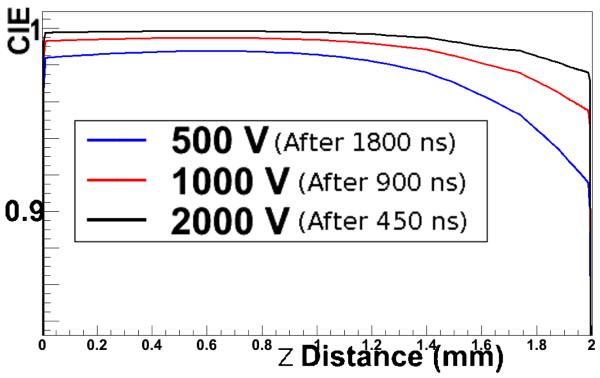
Comparison of the CIE versus *z* for different bias voltages.

Figure 9 shows the CIE versus interaction depth for different values for the bias voltage *ϕ*. The difference is mainly noticeable near the anode, where the contribution is mainly due to holes which, with lower bias voltage, will move slower towards the cathode and hence have more chance to get trapped. However, for the final charge sharing, averaging over all impact points within the 1 × 1 × 2 mm^3^ pixel, the difference is negligible and even with a bias voltage of 500 V the value for the charge sharing is equal as for the case with 2000 V.

## 4 Conclusions

For a CdTe pixel of size 1 × 1 × 2 mm^3^, we found 19.5% and 26% of charge sharing in a 3×3 pixel array with 122 keV and 511 keV gammas respectively, using a threshold of 15 keV. Similar values for the charge sharing were found for different bias voltages 500 V, 1000 V and 2000 V.

## Figures and Tables

**Table 1:** Semiconductor detector material properties (from [8]).

	CdTe
electron mobility *μ_e_*	1100 [cm^2^/Vs]
electron lifetime *τ_e_*	3 × 10^−6^ [s]
hole mobility *μ_h_*	100 [cm^2^/Vs]
hole lifetime *τ_h_*	2 × 10^−6^ [s]
Relative permittivity (*ε*)	10.6
Density	5850 [kg/m^3^]
Resistivity	10^7^ [Ω· m]

## Footnotes

1 The Voxel Imaging PET pathfinder (VIP) project is funded by ERC-FP7.
